# Periapical Lesion: A Single-Sitting Root Canal Treatment

**DOI:** 10.7759/cureus.37597

**Published:** 2023-04-14

**Authors:** Anjali Sharma, Rohit Sharma, Madhurima Sharma, Anushka Jain

**Affiliations:** 1 Conservative Dentistry and Endodontics, Teerthanker Mahaveer Dental College and Research Centre, Moradabad, IND; 2 Prosthodontics and Crown and Bridge, Teerthanker Mahaveer Dental College and Research Centre, Moradabad, IND

**Keywords:** root canal treatment, periapical lesion, non-surgical, metapex, intracanal medicament

## Abstract

The aim of root canal treatment is to disinfect the root canal completely and prevent the progression of any periapical infection. Surgical treatment of periapical lesions is associated with many complications and challenges. This article describes the management of a periapical lesion of the right lower premolar in a single-visit root canal procedure using Metapex. The patient was observed for one week for any incidences of flare-ups.

## Introduction

Periapical lesions may cause severe toothache or may be asymptomatic and are often diagnosed during routine radiographic examination. All periapical lesions should be treated non-surgically initially. Non-surgical treatment involves root canal treatment, for which the rate of success is 86.02% [1]. Some dentists prefer not to perform single-visit root canal treatment in cases involving a periapical lesion as according to their knowledge it can lead to flare-ups [2]. The flare-up is sudden occurrence of severe swelling and pain after a root canal appointment that can lead to an unscheduled visit and requires immediate treatment [3]. Metapex, a widely used intracanal medicament, consists of calcium hydroxide, iodoform, and barium sulphate. The prevalence of *Enterococcus faecalis* is found to be higher in root canal. Also, *E. faecalis* affects the microbial load and size of the periapical lesion [4]. Compared to calcium hydroxide alone, Metapex is found to have better effectiveness against *E. faecalis*. Moreover, Metapex has no known toxicity issues when used in procedures involving periapical extrusion. However, some studies report delayed healing with Metapex [5]. This case report describes a single-sitting root canal procedure involving a periapical lesion managed using Metapex as an intracanal medicament.

## Case presentation

A 23-year-old male patient reported to the Department of Conservative Dentistry and Endodontics with persistent severe pain and no swelling in his lower right back tooth region. On intraoral examination, tooth 44 (right mandibular first premolar) was found to be grossly carious and tender upon percussion. No vestibular obliteration or swelling was noticed. The tooth did not respond to electric or thermal pulp testing. The adjacent teeth showed normal responses to both tests. Periodontal probing did not cause any bleeding. An intraoral periapical radiograph revealed a periapical lesion with ill-defined borders involving the apical aspect of the root of 44. A single-visit root canal treatment was planned.

**Figure 1:**
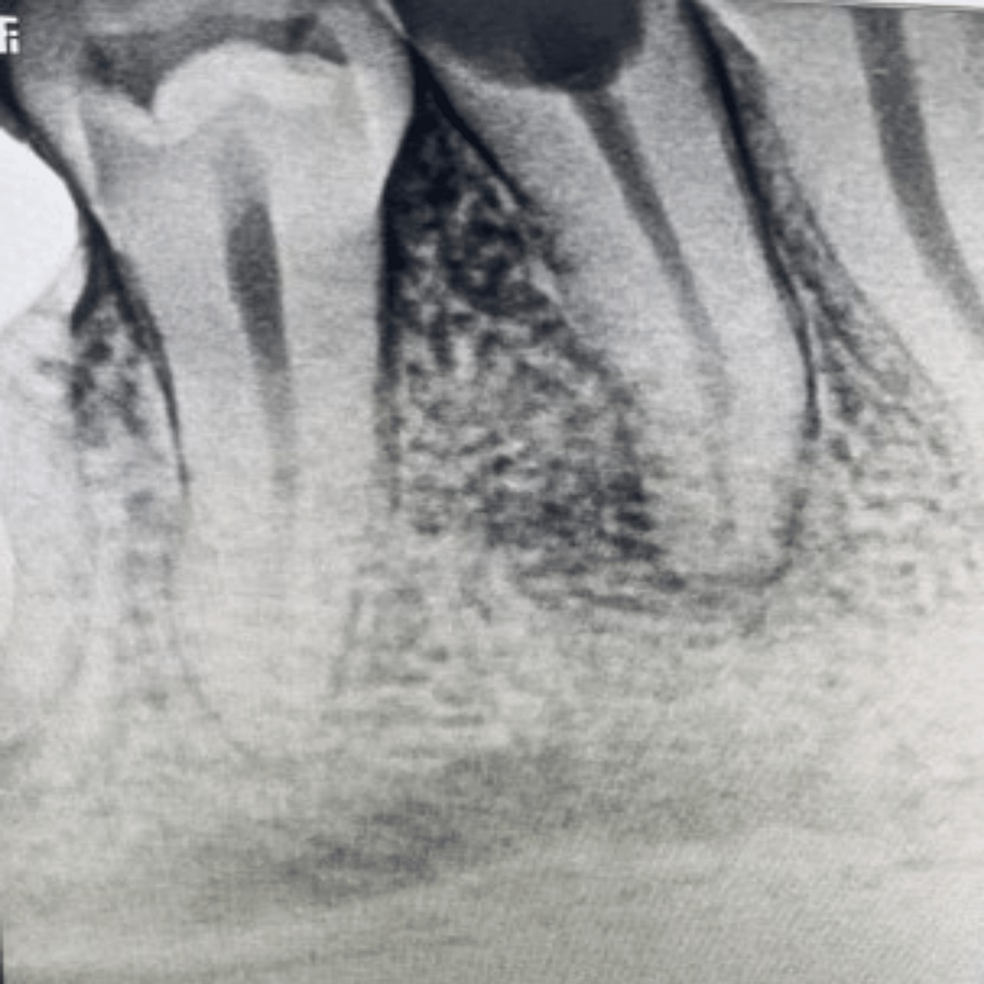
Access opening

**Figure 2:**
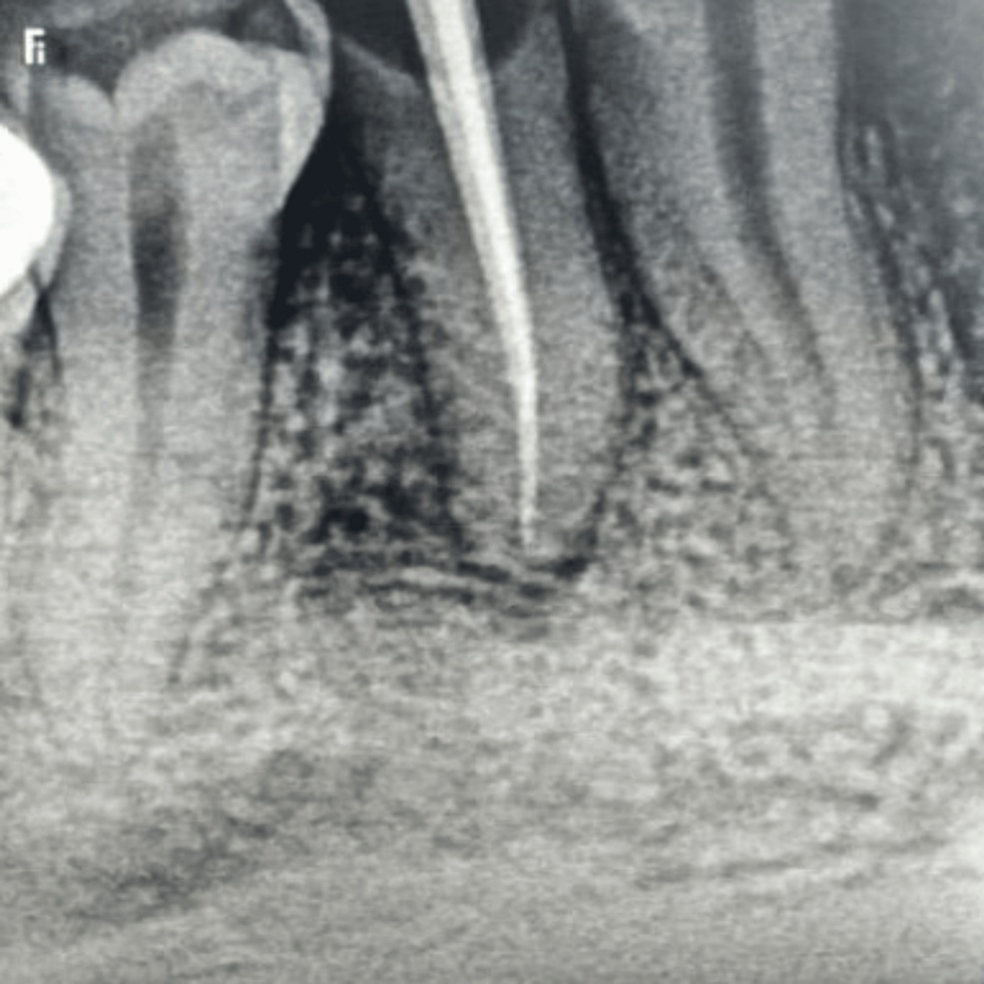
Master cone: F3 in palatal canal and F2 in buccal

**Figure 3:**
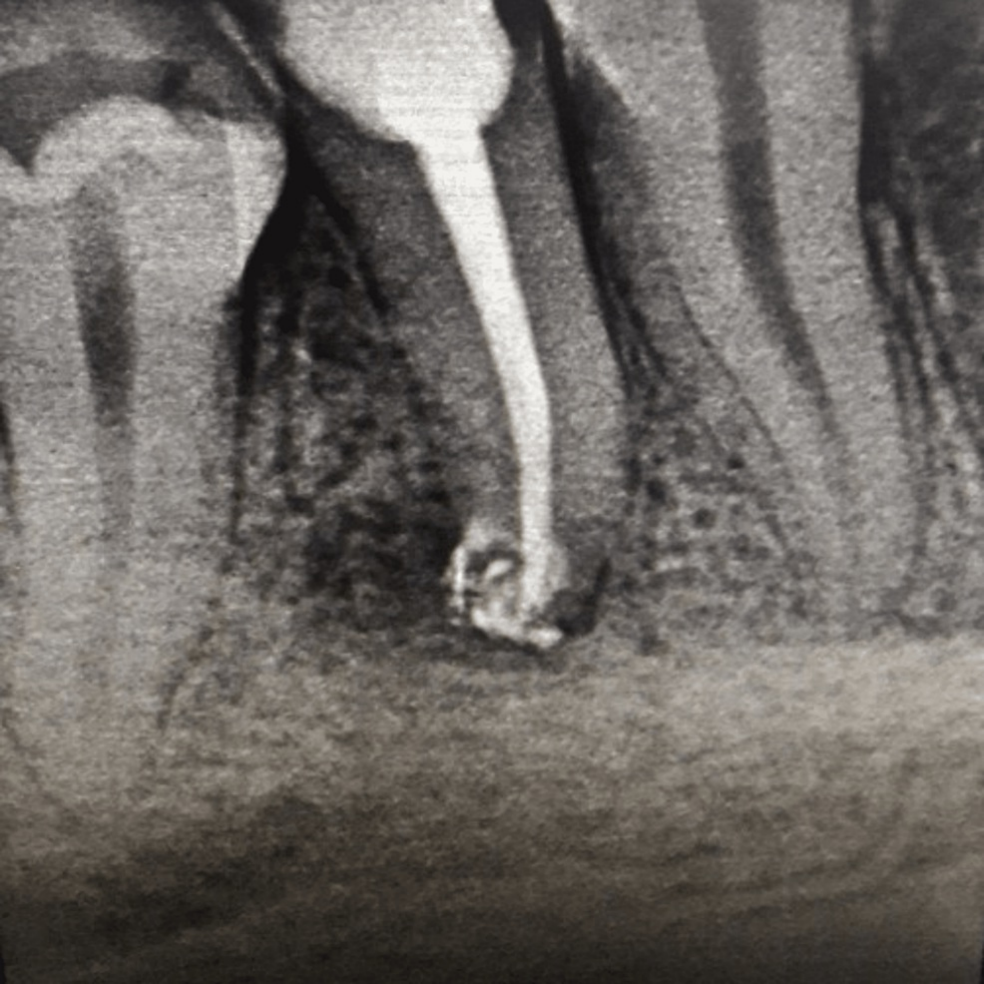
Obturation using gutta-percha, Metapex, and ZOE sealer ZOE: zinc oxide/eugenol

Following access cavity preparation (Figure 1), no pus discharge was noticed. The pulp chamber was initially irrigated with normal saline followed by working length determination. Shaping and cleaning were done using K-File till ISO#15, followed by ProTaper Gold till F3 (Dentsply Sirona, Charlotte, North Carolina, United States) in the lingual canal and F2 in buccal (using ethylene-diamine-tetraacetic acid (EDTA) gel). Only normal saline was used for irrigation in between files. Finally, canals were irrigated with 3% warm sodium hypochlorite, normal saline, and chlorhexidine followed by normal saline. Canals were dried using a paper point. After biomechanical preparation, master cones were verified radiographically (Figure 2) and canals were dispensed with Metapex paste and obturated in the same sitting using F3 and F2 gutta-percha with zinc oxide eugenol sealer (Figure 3).

The patient was kept on antibiotics (Augmentin, Metrogyl), pain killers (Zerodol Sp) and proton pump inhibitor (Pan 40) for three days. He was asked to follow up through telephonic conversion for any incidence of pain and swelling for seven days. The patient had no pain on the first day, and moderate pain on the second and third days. After the fourth day, he did not notice any pain. No swelling was encountered for seven days. The patient reported back after seven days and at one month with no incidence of flare-ups. The patient will be followed up periodically until complete bone fill is evident radiographically.

## Discussion

The first treatment approach for any periapical lesion is root canal treatment that is aimed at reducing the microbial load [6]. Torabinejad et al., in their study, noticed a high rate of success for the non-surgical re-treatment approach in a follow-up of four to six years [7].

Cleaning and shaping with sodium hypochlorite, EDTA, chlorhexidine, warm saline, and Metapex are recommended for infection neutralisation of periapical lesions [8,9]. Metapex is the most successful intracanal medicament for teeth having periapical lesions. It consists of calcium hydroxide as one of its components, which has actions like acid neutralization, anti-inflammatory and inducing cellular differentiation that ultimately leads to the healing of periapical lesions [3]. Metapex is found to be more effective against *E. faecalis* compared to calcium hydroxide alone. Khasawnah et al. assessed the rate of healing of periapical lesions by using Metapex. They concluded that Metapex enhances the healing of periapical lesions [10]. In their study, Galhotra et al. concluded that unintentional periapical extrusion of the Metapex does not have any negative effect on periapical healing [5]. Ayodhi et al. used Metapex for the healing of large periapical lesions. They concluded that Metapex is very beneficial for the healing of periapical lesions [8].

Most dentists renew calcium hydroxide dressing many times for prolonged periods for the healing of such lesions as they are afraid of flare-ups. This is irritating both for the patient and the dentist. Also, it leads to a loss of patient follow-up. Hence, in this study, we used Metapex for a single-sitting root canal of a tooth with a periapical lesion.

Only the incidence of flare-ups is evaluated in this case report. Evaluation of the rate of healing with a longer period of follow-up and a higher sample size is recommended for future studies.

## Conclusions

Single-sitting root canal treatment with Metapex could be considered in the treatment planning of cases of the tooth with periapical lesions in the absence of swelling/exudate. Also, there are minimal chances of flare-ups if Metapex is used in a single sitting for teeth having periapical lesions without any exudate. Healing of teeth with periapical lesions is possible by using Metapex in a single sitting as it is an intracanal medicament that can help in the healing of periapical lesions effectively. Also, non-surgical treatment should always be preferred over a surgical approach to avoid any complications. 
